# Effect of Bleaching and Hot-Pressing Conditions on Mechanical Properties of Compressed Wood

**DOI:** 10.3390/polym14142901

**Published:** 2022-07-17

**Authors:** Le Van Hai, Duc Hoa Pham, Jaehwan Kim

**Affiliations:** 1 Creative Research Center for Nanocellulose Future Composites, Inha University, 100 Inha-ro, Michuhol-ku, Incheon 22212, Korea; levanhai121978@gmail.com (L.V.H.); phamduchoa.tdt@gmail.com (D.H.P.); 2 Pulp and Paper Dept, Phutho College of Industry and Trade, Phongchau, Phuninh, Phutho 290000, Vietnam

**Keywords:** compressed wood, bleaching, lignin content, alpha-cellulose, swelling

## Abstract

This paper reports on multiple stage bleaching and its effect on the mechanical and swelling properties of compressed wood (CW). The natural wood specimen was bleached with NaClO_2_ in five steps and three hot-pressing conditions. Their effects were investigated in morphologies: lignin content, alpha-cellulose content, compression ratio, mechanical properties, swelling and, water contact angle. After compression, the wood specimens became dense and the most porous structures collapsed. The lignin content decreased as the bleaching steps progressed, and the highest alpha-cellulose content was observed at the third bleaching step. This CW showed the best mechanical properties: bending strength was 240.1 ± 35.7 MPa, and Young’s modulus was 23.08 ± 0.89 Gpa. The CW swelling decreased as the bleaching step progressed, and was associated with the density decrease and the compression ratio increase with the bleaching step. The B3 is an optimum bleaching step that accounts for the best mechanical properties, which might be associated with the highest alpha-cellulose content.

## 1. Introduction

Wood is one of the polymer matrix composites nature produces. In total, 4–5 billion cubic meters of wood are harvested annually for various applications such as houses, furniture, construction, ships, bridges, pulp and papers, and many others [1]. Due to its organic cell-type structure, the specific strength and stiffness in certain cases of wood can reach notable values competitive with other construction materials, such as low carbon steel and aluminum alloys [2]. Compressed wood (CW), or densified wood, has been investigated for a long time, and there have been many reported applications of CW, including flooring products [3]. CW can reduce the porosity or void space of the wood and make highly compact wood, leading to better mechanical properties. There are many different approaches for fabricating CW [1,3,4,5,6,7,8,9]: thermo-hydro-mechanical (THM), thermo-mechanical, heat treatment, chemical, surface densification, steam treatment, non-steaming densification, and so on [10,11]. The thermo-mechanical method uses an open hot- press without controlling the humidity and moisture content. Densified wood showed great strength, low price, good stability, and an environmentally friendly character [11].

In CW, lignin removal and the compression condition are important for improving the mechanical properties. Yano’s group bleached wood with sodium chloride and then infiltrated it with phenol-formaldehyde resins [12], such that the tensile strength was improved from 317.4 MPa to 453.7 MPa, and Young’s modulus from 26 GPa to 39 GPa. They reported that the wood specimen’s weight loss is from 14 to 24%, mainly from lignin and hemicellulose. However, they did not report the lignin and cellulose content after bleaching. Song et al., 2018 [13] densified the wood with sodium hydroxide and sodium sulfite bleaching followed by compression at 100 °C and 5 MPa for one day. The CW exhibited a great improvement in the tensile strength from 100 MPa to more than 400 MPa. As the lignin content decreased from 20.8% to around 0.13% by cooking with sodium hydroxide and sodium silicate, the cellulose content reduced from 44.01% to 31.2%. Fang et al., 2012 [6] adopted different temperature and steaming conditions for preparing CW. Using different heating temperatures from 140 °C to 220 °C and the pressure from 4.5 MPa to 9.0 MPa, the tensile strength increased from 50–70 MPa to 150 MPa. Fang et al., 2020 [14] also reported that CW could gain super mechanical properties using sodium hydroxide and anthraquinone: 1.13 GPa of tensile strength and 50–60 GPa of Young’s modulus. The lignin content reached around 1% after several hours.

There has not been any detailed research on the bleaching condition effect on the alpha-cellulose content that influences mechanical properties of CW, although the lignin content effect of CW has been reported. According to previous reports, the lignin content is very broad, from 0.13 to 14%, and the cellulose content of CW was not thoroughly investigated for improving the mechanical properties. Thus, this report focuses on multiple-stage bleaching and its effect on CW’s mechanical and swelling properties. The influence of the lignin and cellulose contents was studied to improve the mechanical properties. Lignin is more hydrophobic than cellulose; thus, non-bleached wood absorbs less water than bleached wood. Due to lignin removal, bleached wood absorbs water easily and becomes unstable during humidity changes. Thus, the swelling properties are investigated with the lignin content and cellulose content. A bleached wood surface can easily absorb water as lignin was partially or mostly removed. However, the waterproof nature of CW is important for its applications. Thus, the water contact angle (WCA) of the prepared CW is measured. Lastly, the density changes and the hot-pressing conditions are also studied to monitor their effect on CW. In order to bleach wood, various bleaching agents or methods are used, such as NaOH, Na_2_SO_3_, NaClO_2_, H_2_O_2_, or UV-assisted bleaching methods. Song et al., 2018 [13] used NaOH and Na_2_SO_3_ under a boiling condition to remove lignin. Xia et al., 2021 [15] used a UV-assisted bleaching method to decolorate wood. NaClO_2_ is popularly used for bleaching wood [12,16,17].

In this research, NaClO_2_ was selected for the bleaching process, and the detailed CW preparation process was explained. The specimens’ morphologies were characterized using field-emission scanning electron microscopy (FESEM). The lignin content, alpha-cellulose content, compression ratio, and swelling ratio were examined, and the three-point-bending (TPB) test was performed to characterize the mechanical properties.

## 2. Materials and Methods

### 2.1. Materials

Pinewood (*Pinus koraiensis*) was purchased from a local market in Korea. The wood specimen size was 150 × 50 × 15 mm^3^, and the grain direction was unidirectional along the longitudinal: 150 mm was the longitudinal direction, 50 mm was the tangential direction, and 15 mm was the radial direction. The wood sample was then dried in an oven drier at 103 °C for several days before the experiment. Sodium chloride (NaClO_2_), sodium acetate (CH_3_COONa), and acetic acid (CH_3_COOH) were purchased from Sigma Aldrich. All chemicals used were analytical reagent grade (purity > 99%) and were used as received.

### 2.2. Methods

#### 2.2.1. Bleaching Process

**Figure 1:**
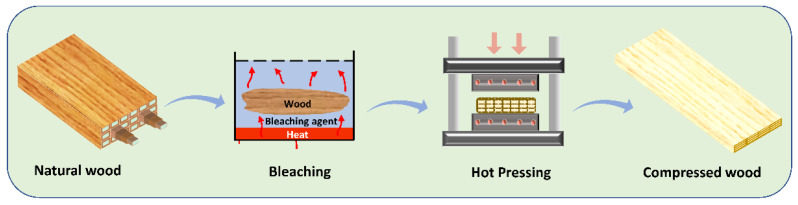
Compressed wood preparation process.

Figure 1 shows the CW preparation process consisting of bleaching and hot pressing. The bleaching condition adopted from Yano et al., 2001 [12] was modified by having multiple steps as follows:

- **Step 1:** 1% of NaClO_2_ was adjusted pH to 4–5 with acetic acid-sodium acetate buffer, then the sample was bleached at 80 °C for 12 h. The bleached wood was washed under running water for around 2 h and kept in a water bath for a day. After washing, the sample was kept in an oven drier at 40 °C for 1–2 days before proceeding to the next bleaching step.
- **Step 2:** In total, 2% of NaClO_2_ was adjusted to a pH of 4–5 with acetic acid-sodium acetate buffer, then the sample was bleached at 80 °C for 12 h. The bleached wood was washed under running water for 2 h and kept in a water bath for a day. After washing, the sample was kept in an oven drier at 40 °C for 1–2 days before proceeding to the next bleaching step.
- **Step 3:** Step 1 was repeated.
- **Step 4:** The sample was kept in 0.1% NaOH suspension for 1 day at room temperature, and then it was washed under running water for 2 h, followed by a water bath for 1 day. After washing, the sample was kept in an oven drier at 40 °C for 1–2 days before processing to the next step.
- **Step 5:** Step 1 was repeated.

The pristine wood specimen was named “NW.” After Step 1 finished, the specimen was called “B1”. Likewise, the specimens were named B3, B4, and B5 after finishing Steps 3, 4, and 5. Since Step 2 was the same as Step 1, except for different NaClO_2_ content, it was not much different, and the specimen was not prepared.

#### 2.2.2. Compressed Wood Preparation

The bleached wood specimens were hot-pressed using a hot-pressing machine (SB3651, HY Industry, Incheon, South Korea) for preparing CW. The hot-pressing machine is a hydraulic-type machine, and the hot-press machine’s maximum hydraulic pressure is limited to 10 MPa. The cylinder area is 0.0195 m^2^. Generally, hot pressing is governed by temperature, pressure, and time. Two temperature conditions were tested for hot pressing: 100 and 150 °C. However, the 150 °C for 8 h was so high that the CW surfaces were always burned. Thus, the temperature was fixed to 100 °C.

The machine’s hydraulic pressure was made only for 5 MPa and 10 MPa, and the hot-pressing time was varied to 4, 8, and 16 h. The pressure and the time variations have six combinations, which are too many cases. According to our preliminary observation, the pressure and time exhibited a similar effect: as the pressure and time increased, the compression increased. Thus, these hot-pressing conditions were accelerated by choosing the following conditions: 5 MPa and 4 h, 10 MPa and 8 h, and 10 MPa, and 16 h. Note that the hydraulic pressure values 5 MPa and 10 MPa correspond to the actual compression pressure of 13 MPa and 26 MPa on the wood specimens. To further increase the compression pressure, one wood specimen area was reduced to 150 × 25 mm^2^ such that the compression pressure was made to 52 MPa. Consequently, the following hot-pressing conditions were made (in terms of actual compression pressure): HP-1: 13 MPa and 4 h; HP-2: 26 MPa and 8 h; and HP-3: 52 MPa and 16 h. The compression ratio was calculated by:

*CR* = (*t_i_* − *t_c_*)/*t_i_* (1)

where *t_i_* is the initial thickness and *t_c_* is the thickness after hot pressing. The prepared specimens were stored in a desiccator (VESD-350, GoodsGood Co., Ltd., Gyeryong-si, Chungcheongnam-do, South Korea) at 15% RH and 23 °C.

### 2.3. Characterization

#### 2.3.1. Morphology

The cross-sectional morphologies of the wood specimens before and after compression were observed using the FE-SEM (H-4000, Hitachi, Tokyo, Japan). The specimens were cut by dipping into liquid nitrogen and breaking them. The specimens were coated with platinum in advance and observed with 10 kV.

#### 2.3.2. Lignin and Alpha-Cellulose Contents

The Klason lignin in the wood was determined by following the TAPPI T 222 om-02 [18]. In total, 15 mL of 72 wt% sulfuric acid was added to a beaker containing 0.5 g of wood sample. The wood samples were stirred with a magnetic stirrer for 2 h at 20 °C. After 2 h reaction time, DI water was added to the 72 wt% sulfuric acids to dilute it to 3 wt% sulfuric acid (TAPPI T 222 om-02). The samples were then boiled in a 3-neck flask for 4 h, and the resultant samples were left to cool in a fume hood overnight and then filtered. After the reaction, the crucible cup 1G3 was used to collect and clean the remaining lignin. The collected samples were dried at 105 °C, and the weight of lignin content was determined by:

*lignin* (%) = *m*/*M* × 100 (2)

where *m* is the weight of lignin and *M* is the oven-dried weight of the specimen.

The alpha-cellulose content was characterized according to Hai et al., 2013 [19]. In brief, a one-gram oven-dried weight of bleached cellulose was treated in 25 mL of 17.5% NaOH at room temperature and mixed for 30 min. Then, 25 mL of deionized (DI) water was added to stop the reaction and then washed through glass filers with DI water, after which point 10 mL of 10% acetic acid was added for 5 min and washed with DI water to neutralize it using a vacuum pump. The washed sample was kept in an oven drier at 105 °C overnight and its alpha-cellulose content was determined.

#### 2.3.3. Mechanical Property

The TPB test was carried out using a tensile test machine (TEST ONE, South Korea) with the TPB test fixture. The distance between the two holding bars was 100 mm, the TPB specimen size was 150 × 10 mm^2^^,^, and the thickness ranged from 2.81 to 6.05 mm depending on different bleaching steps and hot-pressing conditions. The TPB test was carried out at 1 mm/min crosshead speed. The tensile test system automatically calculated the bending strength, strain-at-break, and Young’s modulus from the given dimension from the stress-strain curves. At least five samples were tested for each case. All wood specimens were kept in a humidity-controlled chamber (30% RH and 25 °C). Samples were kept in a humidity and temperature-controlled box. The mechanical properties of the samples were characterized using a tensile test machine (TEST ONE, Siheung-si, Kyunggi-do, South Korea) with a humidity/temperature-controlled chamber.

#### 2.3.4. Swelling Ratio

The swelling test was performed to address the stability of the materials under high moisture content. The CW specimens prepared under HP-3 with different bleaching conditions were placed in the humidity-controlled chamber set to 90% RH and 25 °C for 8 h. The swelling ratio was found by:

Swelling Ratio (%) = 100 × (*t_s_* − *t_i_)*/*t_i_* (3)

where *t_s_* represents the thickness after swelling and *t_i_* is the initial thickness.

## 3. Results and Discussion

### 3.1. Morphology

**Figure 2:**
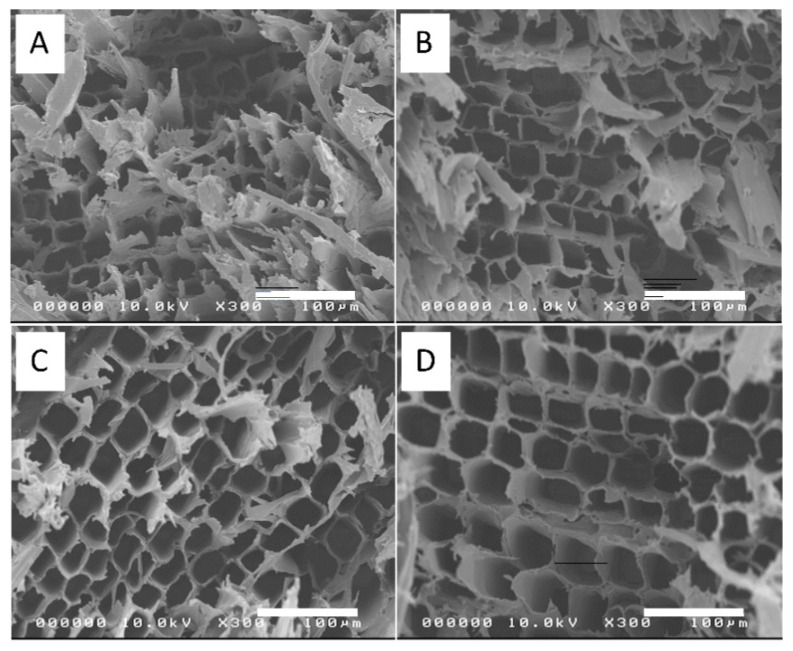
Cross-sectional FESEM images of bleached wood specimens before compression: (**A**) B1, (**B**) B3, (**C**) B4, and (**D**) B5.

**Figure 3:**
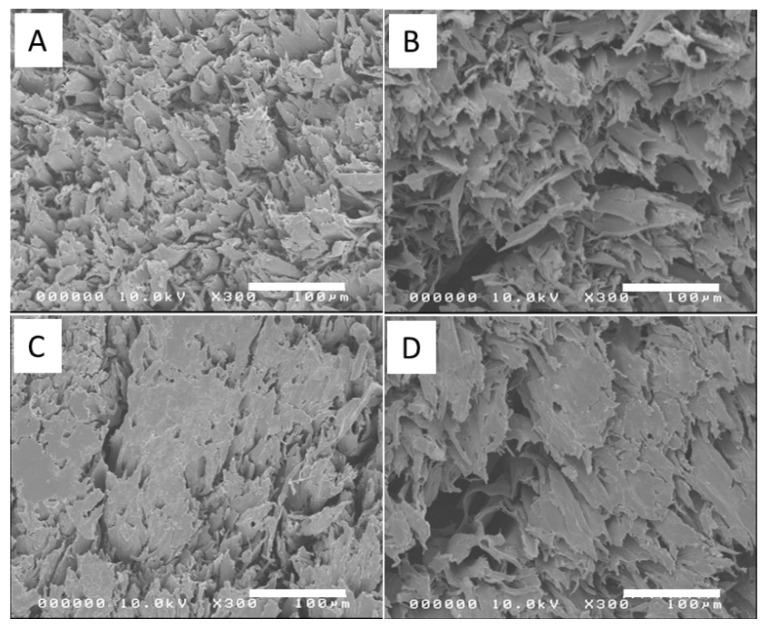

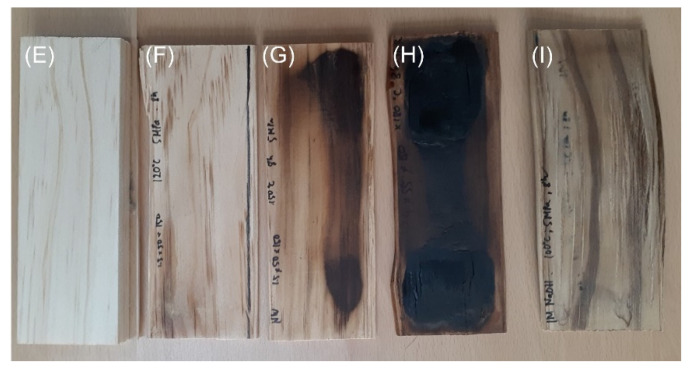
Cross-sectional FESEM images of compressed wood specimens (**A**–**D**): (**A**) B1, (**B**) B3, (**C**) B4, and (**D**) B5; Photographs of wood specimens (**E**–**I**): (**E**) natural wood, (**F**–**H**) compressed natural wood at 120 °C, 150 °C, and 180 °C under 5 MPa, (**G**) compressed B3 at 100 °C and 5 MPa.

Figure 2 shows the cross-sectional FESEM images of the bleached wood specimensthat showed high porosity. As the bleaching steps progressed, more pore structures were observed. Figure 3A–D represents the cross-sectional FESEM images of the CW specimens with HP-3 condition. After compression, the wood specimens became dense and the most porous structures collapsed. The voids in the porous structure were retained in some regions, but most of the voids disappeared. Figure 3E–I shows the photographs of wood specimens for natural wood (E), compressed natural wood with different temperatures at 120 °C, 150 °C and 180 °C under 5 MPa pressure (F-H), and a B3 compressed specimen at 100 °C and 5 MPa. When the hot-pressing temperature was over 100 °C, the specimens were burnt. Thus, 100 °C was chosen for the hot-pressing temperature.

### 3.2. Lignin and Alpha-Cellulose Contents

**Figure 4:**
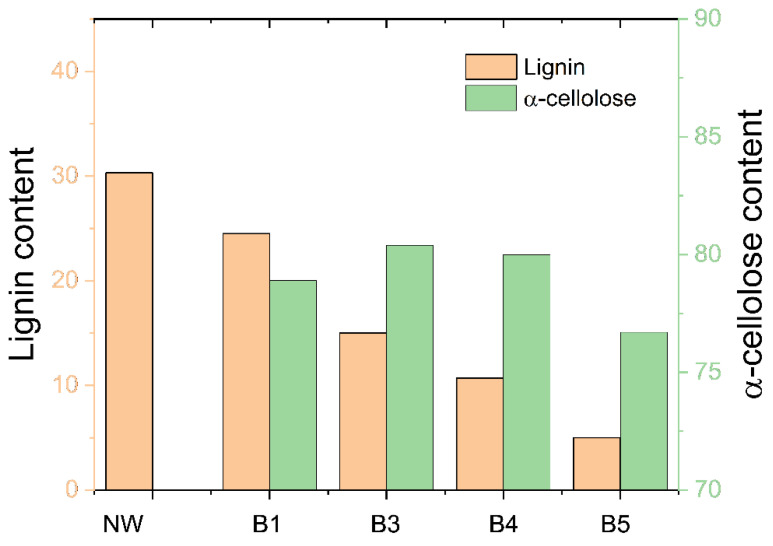
Lignin and alpha-cellulose content with the bleached steps.

Lignin and alpha-cellulose contents were examined under different bleaching conditions, and the results are shown in Figure 4. The lignin content of the natural wood was initially 30.3%, but after bleaching, it decreased to 24.5%, 15.0%, 10.7%, and 5% as the bleaching steps progressed to B1, B3, B4, and B5, respectively. Decreasing the lignin content was natural as the bleaching steps progressed. At the same time, the alpha-cellulose content changed to 78.9%, 80.4%, 80%, and 76.7% for B1, B3, B4, and B5. The B3 and B4 specimens showed higher alpha-cellulose content than the B1. The B5 was the lowest alpha-cellulose content, attributed to over-bleaching. Wood degradation can happen when over-bleaching is done, and thus, the low molecular weight cellulose can be dissolved, leading to lower alpha-cellulose content. The alpha-cellulose content in this research is higher than the holocellulose content reported by several researchers [20,21]. It might be because alpha-cellulose content in this research relied on each bleaching stage using NaClO_2_. One stage bleaching using NaClO_2_ should remain hemicellulose and lignin contents, which were not possible by simple 17.5% NaOH use in the alpha-cellulose analysis.

### 3.3. The Compression Ratio

**Figure 5:**
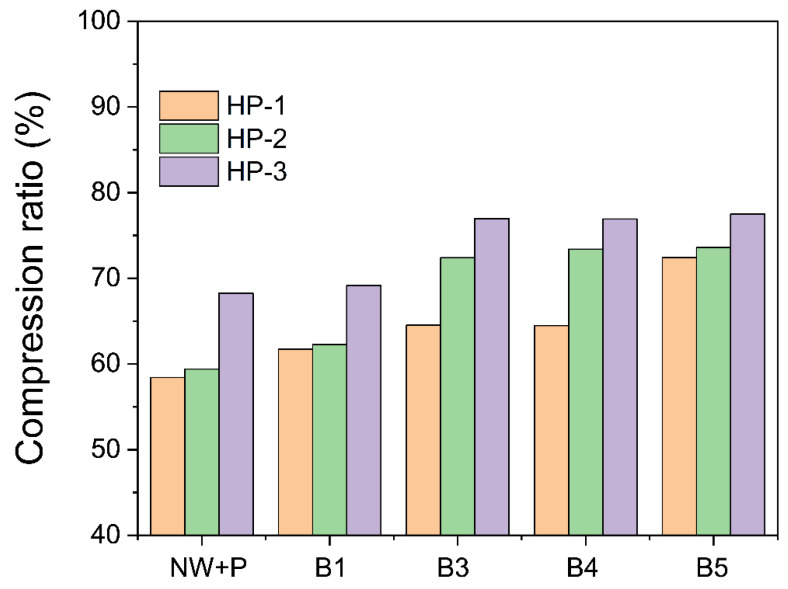
Compression ratios of the wood specimens under different hot-pressing conditions.

The bleached wood specimens were hot-pressured at 100 °C under HP-1, HP-2, and HP-3 conditions. Figure 5 represents the compression ratio to the bleaching and hot-pressing conditions. As the bleaching steps increased, the hot-pressing pressure, the time, and the compression ratio increased. The natural wood specimen had a 58% compression ratio under the HP-1 condition, termed “NW+P”. It was elevated from 61.7% to 72.4% as the bleaching step progressed from B1 to B5. By increasing the hot-pressing pressure and time to HP-3, the compression ratio increased from its initial value of 68.2% to 77.5%. The compression ratio reached over 77% when the bleaching conditions were B3, B4, and B5 under the hot-pressing conditions. It revealed that under the HP-3 condition, the bleaching step over B3 gives the highest compression ratio.

Note that the compression pressure of 52 MPa of the HP-3 condition is just over the compression yield strength of the wood in the transverse direction, 50 MPa [2]. The compression pressure should maintain the yield strength in the transverse direction of the wood to permanently acquire the collapsed deformation. In addition, the hot-pressing time should be long enough to fully collapse the cell wall and hydrogen bond it in the transverse direction. The longer the time, the better the CW’s hydrogen bond might be formed. However, long compression time consumes much energy, which is not economical. Thus, 16 h of HP-3 might be enough for forming CW. As a consequence, HP-3 was an optimum condition for the hot pressing of CW.

### 3.4. Three-Point Bending Test

**Figure 6:**
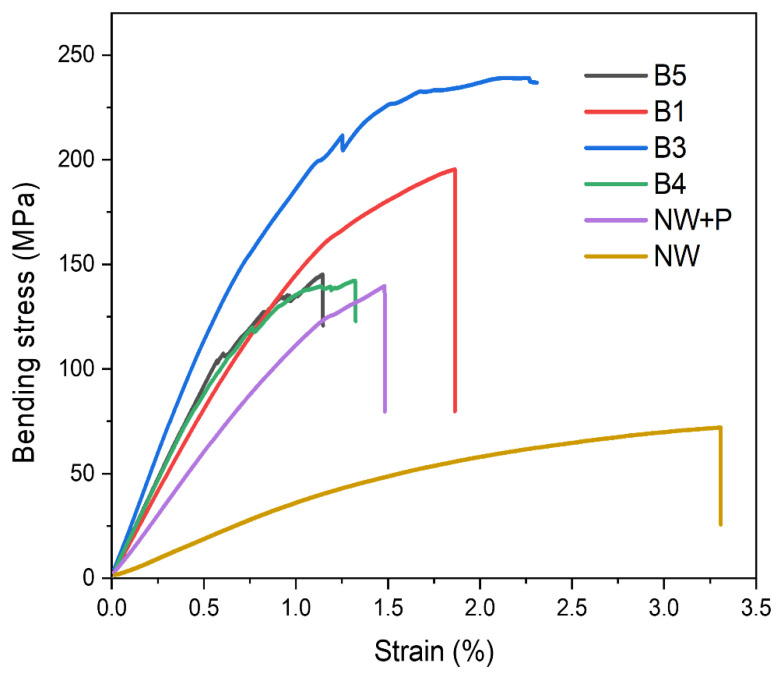
Stress-strain curves of wood specimens under HP-3 hot-pressing conditions (NW: natural wood; NW+P: natural wood hot-pressed; B1-B5: specimens after 1–5 bleaching steps).

The mechanical properties of the wood specimens were investigated using the TPB test. Three hot-pressing conditions (HP-1, HP-2, HP-3) and four bleaching conditions (B1, B3, B4, B5) in addition to the natural wood pressing one (NW+P) were considered. Figure 6 shows the stress-strain curves of the wood specimens according to the bleaching conditions under HP-3 and 100 °C hot-pressing conditions. The original NW specimen was so weak that it was broken at low strength. Its bending strength and Young’s modulus were 74.1 MPa and 3.7 GPa. The compressed NW (NW+P) was better than the NW: the first linear region gives it a Young’s modulus of 12.15 GPa, and the second deformation region was observed near 1.5% strain, which is typical behavior in structural composites because of filament and matrix failures. The bending strength of NW+P was 142.0 MPa. As the bleaching step progressed, the wood specimens increased their Young’s modulus and bending strength. B3’s bleaching condition exhibited 240.1 MPa bending strength and 23.08 GPa Young’s modulus, which indicates that the CW became much tougher than the original NW.

**Figure 7:**
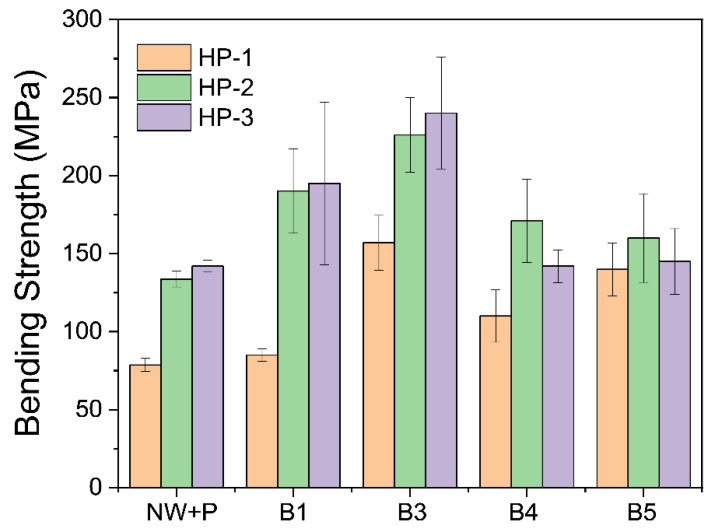
Bending strength of CW specimens prepared by various bleaching and hot-pressing conditions.

Table 1 represents the bending strength, Young’s modulus, and elongation-at-break for all hot-pressing and bleaching conditions in addition to the natural wood hot-pressed case. Figure 7 represents their bending strength trend. The bending strength increased with the bleaching condition up to B3 and decreased after over-bleaching. HP-3 condition exhibited better bending strength than other hot-pressing conditions: HP-1 and HP-2. The highest bending strength of 240.1 MPa was obtained from HP-3 hot-pressing and B3 bleaching conditions.

**Figure 8:**
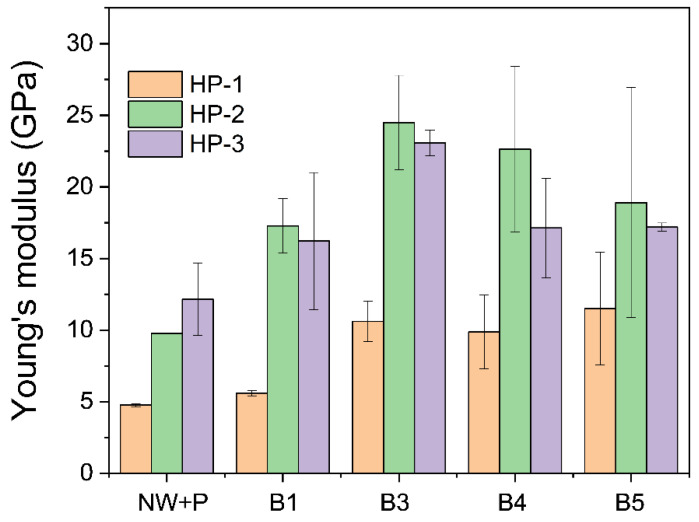
Young’s modulus of CW specimens prepared by various bleaching and hot-pressing conditions.

Figure 8 shows the Young’s modulus trend of wood specimens. Young’s modulus increased with the bleaching steps, and after B3, it decreased. The HP-2 condition exhibited a better Young’s modulus than the other two conditions: HP-1 and HP-3. The maximum Young’s modulus of 24.49 GPa was obtained at HP-2 hot-pressing and B3 bleaching conditions. Long hot-pressing time might result in an annealing effect such that it decreases Young’s modulus and increases the strain-at-break and bending strength.

In this paper, the highest mechanical properties of CW were observed at the B3 bleaching condition, where the lignin content was 15.0%. Further proceeding with the bleaching step can increase the lignin removal, decreasing the mechanical properties due to decreased cellulose content. Thus, an optimum bleaching step is B3, and further bleaching is not recommended. Song et al., 2018, reported the best mechanical properties when the lignin content was 11.3% [13]. Fang et al., 2020 [14], made 3% lignin content to achieve good mechanical properties. The lignin content that achieves the best mechanical properties may differ depending on the chemicals and hot-pressing conditions. In terms of mechanical development, the bleached CW shows good mechanical properties. The strength of NW, 74 MPa reached over 240 MPa after compression, which is over three times high. According to Song et al. [13], the NW strength of 50 MPa reached over 500 MPa depending on the bleaching treatment and multilayers compression. Fang et al. [14] reported that the bending strength of NW, about 50 to 70 MPa, can be reached to 120–140 MPa, depending on the thermal treatment from 160 °C to 220 °C, whereas Yano et al., 2001, produced transparent wood with mechanical properties from 317 MPa to 453 MPa, which is combined by compression and filler reinforcement. Paril et al. [4] reported that the TPB strength of natural wood and compression wood was from 96 to 160 MPa. It shows that by bleaching and compression, the bending strength of bleached wood is much better than normal compression wood. This research presented that compression wood can reach 240 MPa with the three stages of bleaching treatment. As seen below, parts of Table 1 show mechanical properties of the previous reports for densified wood samples.

### 3.5. Swelling

**Figure 9:**
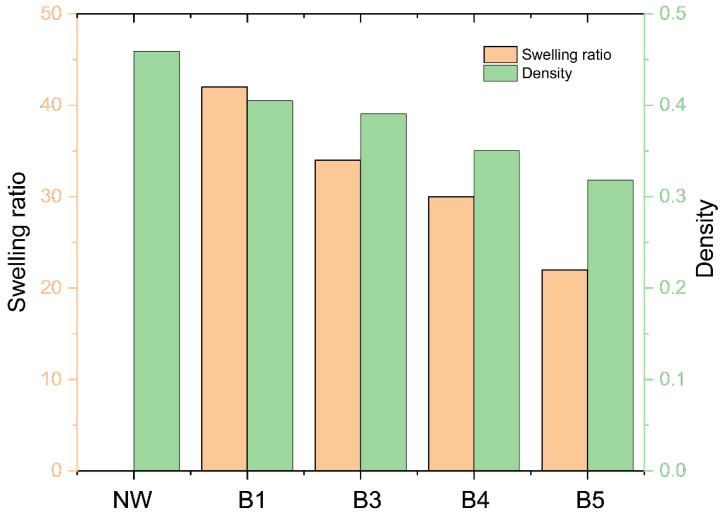
The swelling ratio of CW specimens and density change with bleaching steps.

The swelling test was performed to address the stability of the materials under high moisture content. The CW specimens prepared under HP-3 with different bleaching conditions were placed in the humidity-controlled chamber set to 90% RH and 25 °C for 8 h. The CW swelled after bleaching step 1 (swelling ratio = 42%) and monotonically decreased to 20% as the bleaching step progressed, as shown in Figure 9. The swelling of woods varies and is closely related to the relative chemical content, morphological structures, temperature, and relative humidity [22]. The relationship between the carbohydrates and the other components (e.g., lignin and extractives) on the swelling characteristics of wood have already been well-documented. It has also been reported that the swelling of compressed wood increased as the density increased [23]. Figure 9 also shows that the density decreases as the bleaching step progresses. Note that the density was measured before compression. The longer the bleaching steps, the higher the lignin and hemicellulose removal, leading to a highly porous wood structure and low density of the bleached wood. The swelling decrease might be mainly associated with the density decrease, and the compression ratio increase as the bleaching step continued. Although B5 has the lowest lignin content (see Figure 4), it exhibited the lowest swelling ratio.

The weight and density changes of the specimens were measured before and after compression. Table 2 shows the results. As one can see, the weight significantly decreased as the bleaching step progressed, which resulted in the original density decrease. The weight decrease was associated with the lignin and hemicellulose removal by the continued process. Note that the hot pressing significantly increased the CW densities 3–4 times more than the original densities. As the hot-pressing condition changed to HP-1, HP-2, and HP-3, they increased. The CW density ranged between 1.0–1.2 under HP-1 but increased to 1.1–1.4 and 1.3–1.7 under HP-2 and HP-3. The CW density change by the bleaching steps increased as the hot-pressing condition changed from HP-1 to HP-2 and HP-3. The weight decrement with the bleaching steps mitigated the volume decrement by the compression as the bleaching steps progressed. Thus, the CW density change by the bleaching steps was not significant.

**Figure 10:**
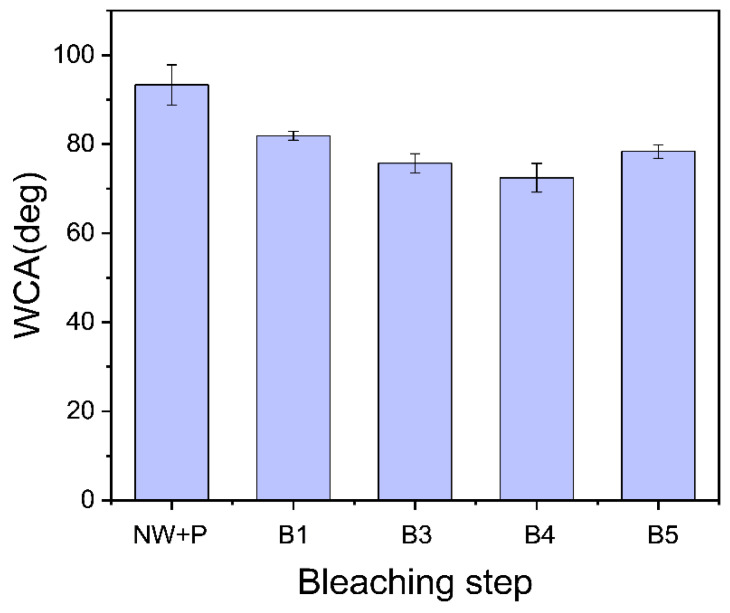
Water contact angles of CW specimens.

The water contact angle (WCA) of the heat-treated CW increases the hydrophobic properties [24,25]. The authors indicated the effects of different heat treatments and compacted surfaces of CW. However, in this research, only one heat-compression condition was carried out, limiting the heat treatment effects. Furthermore, different bleaching treatment leads to different effects on WCA of CW. Longer bleaching times and multiple bleaching stages raise the higher OH groups exposed to the surface of wood specimens. They lead to decreased hydrophobicity. The WCA of CW specimens was measured to investigate their water-proof behavior. Figure 10 shows the WCA results. As the bleaching process continued, the WCA decreased to 72.5° at B4 and bounced back at B5. Surface morphologies and lignin content can influence the WCA. Hemicellulose is the most hygroscopic polymer in wood, followed by lignin and crystalline cellulose. The WCA decreased as the lignin content decreased with the bleaching step (see Figure 4). The slightly bounced back WCA at B5 might be associated with the smooth surface morphology of B5, resulting in the highest compression ratio.

## 4. Conclusions

Compression wood was prepared by removing lignin with multiple steps of NaClO_2_ bleaching, and the bleaching effects were investigated in terms of morphologies, lignin content, alpha-cellulose content, compression ratio, mechanical properties, swelling, and water contact angle. After compression, the CW densities increased, and the most porous structures collapsed. The lignin content decreased as the bleaching steps progressed, and the highest alpha-cellulose was observed at the B3 and B4 bleached specimens. The compression ratio reached over 77% when the bleaching condition was over B3 under the HP-3 hot-pressing condition, which was just over the compression yield strength of the wood in the transverse direction. The B3 CW showed the best mechanical properties: bending strength 240.1 ± 35.7 MPa and Young’s modulus 23.08 ± 0.89 GPa. Over the B3 step, the mechanical properties decreased due to over-bleaching. The swelling of CW decreased as the bleaching step progressed, which was associated with the density decrease and the compression ratio increase with the bleaching step. The water contact angle decreased to 72.5° at B4 as the bleaching process continued. The B3 is an optimum bleaching step that accounts for high mechanical properties, which might be associated with high alpha-cellulose content.

Bleaching treatment with hot pressing can improve the mechanical properties of CW, which is applicable for environmentally friendly structures such as concrete reinforcement. However, the moisture absorption and water swelling behaviors need to be improved, and chemical inertness and thermal and sound absorbing behaviors need to be investigated.

## Figures and Tables

**Table 1:** Mechanical properties of CW specimens prepared by various bleaching and hot-pressing conditions.

Hot-Pressing Condition	Bleaching Condition	Bending Strength (MPa)	Young’s Modulus (GPa)	Elongation-at-Break (%)
Natural wood		74.1 ± 2.9	3.7 ± 0.06	3.23± 0.05
HP-1 (13 MPa and 4 h)	NW±P	78.7 ± 4.3	4.76 ± 0.11	2.10± 0.10
	B1	85.0 ± 3.9	5.60 ± 0.19	1.90± 0.24
	B3	157.0 ± 17.6	10.60 ± 1.42	2.10 ± 0.24
	B4	110.0 ± 16.8	9.88 ± 2.58	1.30± 0.27
	B5	140.1± 16.9	11.50 ± 3.94	1.50± 0.16
HP-2 (26 MPa and 8 h)	NW±P	133.7 ± 5.1	9.76 ± 0.02	1.60 ± 0.01
	B1	190.1 ± 27.0	17.27 ± 1.90	1.48 ± 0.16
	B3	226.0 ± 23.9	24.49 ± 3.30	1.70 ± 0.17
	B4	171.1 ± 26.7	22.64 ± 5.8	1.18 ± 0.60
	B5	160.0 ± 38.4	18.90 ± 8.04	1.35 ± 0.05
HP-3 (52 MPa and 16 h)	NW±P	142.0 ± 3.9	12.15 ± 2.54	1.54 ± 0.01
	B1	195.0 ± 52.2	16.22 ± 4.78	1.62 ± 0.16
	B3	240.1 ± 35.7	23.08 ± 0.89	1.97 ± 0.17
	B4	142.0± 10.5	17.14 ± 3.48	1.28 ± 0.60
	B5	145.0 ± 21.0	17.20 ± 0.28	1.23 ± 0.05
Song et al. [13]	Partial lignin removal by NaOH, Na_2_SO_3_	549	42	1.3
Fang et al. [14]	alkaline sulfite-anthraquinone-methanol cooking	1000	60.2	3
Paril et al. [4]	chemical modification of gaseous ammonia	160	16	-

**Table 2:** Density changes of specimens under various bleaching steps and hot-pressing conditions.

Specimen	Weight (g)	Original Density (g/cm^3^)	Density after HP-1 (g/cm^3^)	Density after HP-2 (g/cm^3^)	Density after HP3 (g/cm^3^)
NW	22.70	0.459	1.104	1.131	1.447
B1	18.05	0.405	1.059	1.074	1.314
B3	18.65	0.390	1.101	1.415	1.695
B4	15.90	0.350	0.986	1.317	1.518
B5	15.05	0.318	1.154	1.205	1.413

## Data Availability

Not applicable.
